# Editorial: Molecular Magnets

**DOI:** 10.3389/fchem.2019.00229

**Published:** 2019-04-11

**Authors:** Matilde Fondo, Albert Escuer, Juan Manuel Herrera

**Affiliations:** ^1^Departamento de Química Inorgánica, Facultade de Química, Universidade de Santiago de Compostela, Santiago de Compostela, Spain; ^2^Departament de Quimica Inorgànica i Orgànica and Institut de Nanociencia i Nanotecnologia (IN^2^UB), Universitat de Barcelona, Barcelona, Spain; ^3^Departamento de Química Inorgánica, Facultad de Ciencias, Universidad de Granada, Granada, Spain

**Keywords:** molecule-based magnet, single molecular magnet, modeling and theory, exchange and magnetic interaction, lanthanoids and transition metals

Since the discovery of single molecule magnetism in a Mn_12_ complex in 1993, the research on this field has received growing attention. This is mainly due to the great advantages that the molecular magnets would have in storage and data processing with respect to the classic magnets. Moreover, on basis on their quantum size effects, other applications beyond high-density information storage have been proposed, such as spintronics or qubits for quantum computing. This special issue in Molecular Magnets compiles 7 articles on the latest advances in this research topic.

Firstly, an article from De et al. illustrates how the spin crossover (SCO) can be light or thermally induced in a series of mononuclear(II) iron complexes, and how the SCO affects the optical properties of these compounds. Both magnetic and optical properties are rationalized on the basis of DFT calculations.

Then, the article by Fondo et al. deepen in the magnetic behavior of dysprosium and terbium complexes with phosphine and arsine oxides as ligands. The comparison of the results described herein with those previously reported with cyclohexylphosphine oxide clearly shows that the steric hindrance caused by the phosphine substituents, as well as by the auxiliary ligands, is the fundamental factor that determines the structure of the complex, and, accordingly, its magnetic properties.

Maniaki et al. review the 3d-metal clusters that acts as single molecule magnets (SMMs). This article, which is focused on the synthetic methodology, provides a comprehensive perspective of how the “trial and error” methods have given way to much more rational synthetic alternatives in the search for polynuclear 3d-metal molecular magnets.

Díaz-Ortega et al. present a bifunctional chiral/SMM Dy_2_ complex, increasing the small number of complexes where both SMM behavior and chirality coexist. They compare the U_eff_ of this SMM with similar reported non-chiral complexes, and the obtained results are justified by means of DFT calculations, thus contributing to understand the factors that affect the anisotropic energy barrier in complexes with DyN_2_O_6_ cores.

Lefeuvre et al. present a new binuclear Dy(III) complex with a redox active ligand, with six crystallographically independent Dy^III^ centers. The magnetic analysis of the complex shows that it displays multi-component field-induced single molecule magnet behavior, and this behavior is explained based on DFT calculations. The compound also shows a redox activity that converts it into an ideal candidate for materials exhibiting redox switching of magnetic properties.

The paper by Perfetti et al. goes in depth in the study of the anisotropy of a related heterooctanuclear Cr_4_Dy_4_ single molecule magnet, and they show how single crystal magnetometry and cantilever torque magnetometry can help to elucidate the orientation and magnitude of the magnetic anisotropy of each paramagnetic ion in a heteronuclear cluster.

Finally, Charalambous et al. deal with the rational synthesis and magnetic properties of several giant heteronuclear (up to 40 metal center) Mn_x_M_y_ (M = Ni or Co) complexes, which are among the largest heterometallic Mn-containing clusters. The work clearly shows the importance of the anisotropy in the single molecular magnet behavior, given that the nanosized Mn_36_Ni_4_ aggregate, which present the second highest value of ground state reported for a heterobimetallic cluster (*ca*. 26), is not a SMM, while the Mn_32_Co_8_ analog shows slow relaxation of the magnetization at T < 3.5 K.

The editors hope that the articles compiled in this publication will be worthwhile for researchers working on the field of molecular magnetism, and that they will contribute to improving the knowledge and properties of molecular magnets. For this reason, we would like to thank the effort carried out by all those who have made it possible for this special issue to see the light: authors, co-authors, reviewers, and the Frontiers in Chemistry development team.

## Author Contributions

All authors listed have made a substantial, direct and intellectual contribution to the work, and approved it for publication.

### Conflict of Interest Statement

The authors declare that the research was conducted in the absence of any commercial or financial relationships that could be construed as a potential conflict of interest.

